# Protocol for quantifying mitotic progression in adherent and suspension cells by live-cell imaging

**DOI:** 10.1016/j.xpro.2026.104899

**Published:** 2026-10-13

**Authors:** Siamak A. Kamranvar

**Affiliations:** 1 Division of Cancer and Genetics, School of Medicine, Cardiff University, CF14 4XN Cardiff, UK

**Keywords:** Cell Biology, Cancer, Microscopy

## Abstract

Live-cell imaging is used to monitor mitotic progression in adherent cells; however, its application to suspension cells remains limited. Here, we present a protocol for quantifying mitotic progression in adherent and suspension cells by monitoring chromatin condensation using the live-cell DNA probe SPY-DNA. We describe steps for cell synchronization, cell plating, SPY-DNA labeling, and live-cell imaging. We then detail procedures for surface plot visualization and intensity profile analysis. This approach enables direct comparison of mitotic progression between adherent and suspension cells.

For complete details on the use and execution of this protocol, please refer to Kamranvar et al.^1^

## Highlights

• Synchronization of adherent cells for live-cell imaging of mitotic progression
• Monitoring chromatin condensation using the live-cell DNA dye SPY-DNA
• Quantification of mitotic entry, mitotic exit, and mitotic duration
• Comparison of mitotic progression in adherent and suspension cells

## Abstract (editor highlights)

Publisher’s note: Undertaking any experimental protocol requires adherence to local institutional guidelines for laboratory safety and ethics.

## Graphical abstract

**Figure:**
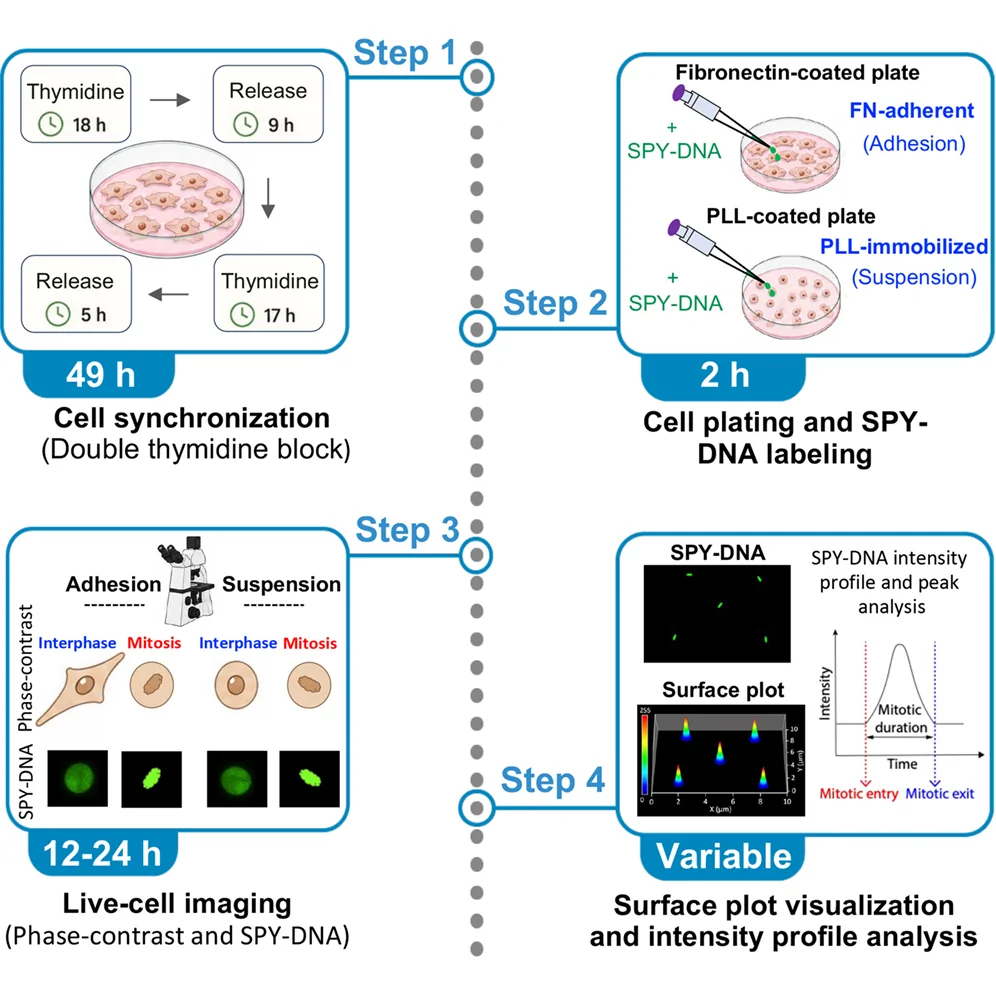

## Before you begin

This protocol describes the quantification of mitotic progression in hTERT-immortalized BJ fibroblasts cultured under adherent and suspension conditions using live-cell imaging. In this protocol, suspension cells refer to anchorage-deprived cells immobilized on poly-L-lysine (PLL) for live-cell imaging. PLL provides sufficient immobilization for longitudinal tracking while minimizing integrin-mediated adhesion signaling and allowing cells to retain a rounded morphology characteristic of suspension cells.^1^ The workflow combines cell synchronization by double thymidine block, SPY-DNA labeling,^2^ and fluorescence intensity analysis to quantify mitotic entry, mitotic exit, and mitotic duration. Although optimized for BJ fibroblasts, this protocol can be adapted to other adherent and suspension cell lines that are compatible with live-cell DNA dyes.

Cell synchronization increases the proportion of cells entering mitosis simultaneously, thereby improving the efficiency of live-cell imaging. In this protocol, a double thymidine block synchronizes cells in early S phase, allowing synchronized progression through G2 and into mitosis following release.^3^ Synchronization in early S phase provides a practical imaging window for monitoring mitotic entry and progression. By contrast, synchronization in G1 may require prolonged imaging before mitotic entry, increasing the risk of photobleaching, whereas synchronization in late G2 may provide only a narrow imaging window and increase the likelihood of missing mitotic entry.^4^

### Validation of adherent and suspension culture conditions

Before performing live-cell imaging, verify that the adherent and suspension culture conditions have been established successfully. Integrin-mediated adhesion can be assessed by western blot analysis of phosphorylated focal adhesion kinase (pFAK-Y397) as described by Kamranvar et al.^1^ Adherent cells cultured on fibronectin (FN) exhibit robust phosphorylation of FAK, whereas PLL-immobilized suspension cells show nearly complete loss of integrin signaling, supporting the successful establishment of an anchorage-deprived culture condition (Figures 1A–1D). Western blotting was performed using antibodies against pFAK (Y397), FAK and β-actin (Figure 1B), followed by HRP-conjugated secondary antibodies. The details of the antibodies are provided in the key resources table.

**Figure 1:**
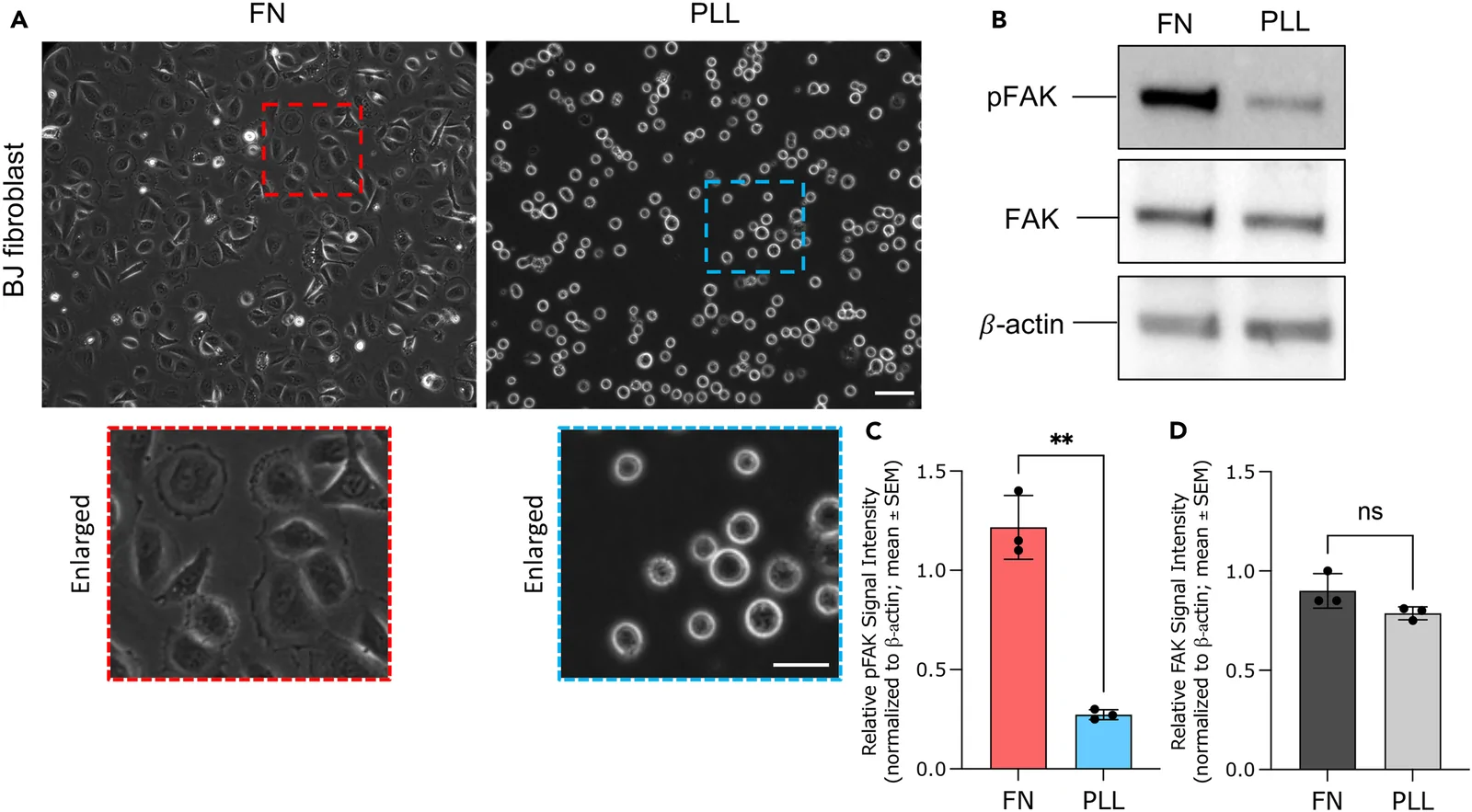
Validation of adherent and PLL-immobilized suspension culture conditions (A) Representative phase-contrast images of hTERT-immortalized BJ fibroblasts cultured on FN-coated plate or PLL-coated plate under anchorage-deprived (suspension) condition. Dashed boxes indicate the regions shown at higher magnification below. Scale bars, 50 μm (main images) and 25 μm (enlarged images). (B) Representative western blot analysis showing phosphorylated focal adhesion kinase (pFAK-Y397), total FAK and β-actin (loading control) in BJ fibroblasts cultured under adherent (FN-coated) and suspension (PLL-coated) conditions across three independent replicates. (C) Quantification of pFAK signal intensity normalized to β-actin. ∗∗ p < 0.01. (D) Quantification of total FAK signal intensity normalized to β-actin. Bars represent mean ± SEM of three independent experiments (*n* = 3). High pFAK signal in FN-adherent cells indicates active integrin signaling, whereas PLL immobilization significantly (*p* = 0.0083) reduces pFAK-Y397 without significantly altering total FAK abundance (ns, not significant, *p* = 0.1398). The signal intensities of pFAK, FAK, and β-actin bands were measured using Image Lab Software. Background intensity was subtracted from each band before normalization of pFAK and total FAK signal intensities to β-actin.

Before beginning the protocol, prepare the following materials and culture conditions.

1. Adherent culture.
  a. Coat 6-well plates with at least 1 ml of FN solution (40 μg/mL in ddH2O), ensuring that the entire well surface is covered.
  b. Incubate the plate for 2 h at 37°C.
  c. Remove the coating solution and wash the wells twice with prewarmed PBS before seeding the cells.
2. Suspension culture.
  a. Coat 6-well plates with at least 1 ml of PLL solution (PLL; 0.01%), ensuring complete coverage of the well surface.
    ***Note:*** PLL was used to gently immobilize suspension cells during the short-term live-cell imaging. Under this condition, cells retained a rounded morphology (Figure 1A) and suspension-like behavior, with integrin-mediated pFAK signaling substantially reduced compared with the FN-coated condition (Figure 1B–1D). For additional recommendations on PLL coating, see the manufacturer’s instructions.
  b. Incubate the plate 12 h at 4°C.
  c. Remove the PLL solution and wash the wells twice with prewarmed PBS before seeding the cells.
    ***Note:*** Poly-D-lysine (PDL) may be considered as an alternative for longer-term applications because of its greater resistance to enzymatic degradation. Agarose (0.8%) or methylcellulose (0.8%) can also be used to generate suspension culture conditions.^5^
3. Warm complete culture medium (DMEM containing 10% FBS, penicillin [100 U/mL], and streptomycin [0.1 mg/mL]), PBS and trypsin-EDTA to 37°C before use.
4. Allow the SPY-DNA solution to reach 18–25°C before use. Protect SPY-DNA from light throughout the experiment.
5. Equilibrate the live-cell imaging chamber to 37°C with 5% CO_2_ before image acquisition.

***Note:*** For parallel imaging of FN- and PLL-coated wells, coat the wells with PLL for 12 h and coat the FN wells the following day. Incubation of the PLL-coated wells at 37°C does not affect PLL coating performance.

**CRITICAL:** Ensure that the temperature and CO_2_ concentration have stabilized before starting image acquisition to minimize focus drift and maintain normal cell-cycle progression.

### Innovation

Conventional live-cell analysis of mitotic progression often relies on characteristic morphological changes, such as cell rounding and spreading, making it unsuitable for suspension cells, which remain rounded and highly mobile throughout the cell cycle.^6^ This protocol overcomes these limitations by quantifying mitotic progression through monitoring chromatin condensation and decondensation dynamics using the live-cell DNA dye SPY-DNA.^7^ Following release from a double thymidine block, chromatin condensation at mitotic entry generates a bright, compact SPY-DNA fluorescence signal that produces a distinct peak in the surface intensity plot, whereas chromatin decondensation during mitotic exit is accompanied by a decrease and disappearance of this peak.

A second innovation is the integration of fluorescence intensity-based surface plot analysis with live-cell imaging, providing a quantitative and morphology-independent approach for quantifying mitotic entry, mitotic exit, and mitotic duration. In addition, gentle immobilization of suspension cells on PLL-coated plates minimizes cell movement during long-term imaging while maintaining a rounded, suspension-like morphology (Figure 1A). Together, these features enable direct comparison of mitotic progression between adherent and suspension cell populations under identical imaging conditions.

## Key resources table

**Table:** 

REAGENT or RESOURCE	SOURCE	IDENTIFIER
**Antibodies**		

pFAK (pTyr397) (1/1000 dilution)	Invitrogen	44624G
FAK (1/1000 dilution)	BD Biosciences	610087
β-actin (1/5000 dilution)	Abcam	ab6276
HRP-conjugated donkey anti-rabbit (1/4000 dilution)	Amersham (Cytiva)	NA9340V
HRP-conjugated sheep anti-mouse (1/4000 dilution)	Amersham (Cytiva)	NA9310V

**Chemicals, peptides, and recombinant proteins**		

DMEM (Dulbecco’s Eagle’s medium)	Gibco	11995-065
Fetal bovine serum (FBS)	Saveen & Werner	FB-1090–500
Trypsin + EDTA	Gibco	3624-01
SPY-DNA	Spirochrome	SPY-505-DNA
SPY-Tubulin	Spirochrome	SPY-555-tubulin
Thymidine	Sigma-Aldrich	T1895
PBS without Ca^2+^ and Mg^2+^	SVA, Uppsala	992420/1000
Fibronectin (FN)	Sigma-Aldrich	F0895
Poly-L-lysine (PLL)	Sigma-Aldrich	P9155
Penicillin/Streptomycin	Gibco	10378016

**Experimental models: Cell lines**		

hTERT-immortalized BJ fibroblasts	Ludwig Institute, Uppsala	RRID: CVCL_3653

**Software and algorithms**		

NIS-Elements AR software	Nikon	Version 4.30.02
Image Lab Software	Bio-Rad	Version 6.1.0

**Other**		

Nikon Eclipse Ti-U inverted microscope	Nikon	Eclipse Ti-U
Andor Zyla 5.5 PLUS sCMOS camera	Oxford Instruments	Andor Zyla 5.5
S Plan Fluor ELWD 20X Ph1 ADM objective lens	Nikon	MRH48230
SOLA SE fluorescence light source	Lumencor	80-10247
Six-well plate, Clear polystyrene	Thermo Fisher Scientific	150239
Stage-top environmental chamber	Okolab	N/A
Tissue culture dish (60-mm)	Sigma-Aldrich	CLS430166

## Step-by-step method details

### Cell synchronization

**Timing: 49 h**

Synchronize BJ fibroblasts using a double thymidine block to enrich the population for cells progressing synchronously toward mitosis.

1. Seed 3 × 10^5^ hTERT-immortalized BJ fibroblasts in a 60-mm tissue culture dish containing complete DMEM and incubate 12 h at 37°C in 5% CO_2_.
2. Add thymidine to a final concentration of 2 mM (100 mM stock solution prepared in ddH_2_O) and incubate for 18 h.
3. Wash the cells twice with prewarmed PBS and once with complete DMEM.
4. Release the cells into the fresh complete medium for 9 h.
5. Add thymidine again to a final concentration of 2 mM and incubate at 37°C in 5% CO_2_ for 17 h.
6. Wash the cells twice with prewarmed PBS and once with complete medium.
7. Incubate the synchronized cells in fresh complete medium for 5 h before trypsinization.

***Note:*** The double-thymidine synchronization procedure takes 49 h from the start of the first thymidine treatment to the end of the 5 h release period (18 h + 9 h + 17 h + 5 h). The initial 12 h culture before the first thymidine treatment is not included in this 49 h period. Following release from the second thymidine block, cells were incubated for 5 h to allow progression from S phase towards G2/M.

**CRITICAL:** Perform all washing steps during synchronization gently to avoid detaching the cells and use prewarmed solutions to maintain normal cell-cycle progression.

**Pause point**: No pause point is recommended after initiation of cell synchronization because the timing of the thymidine block and release steps is critical for synchronization.

### Preparation of adherent and PLL-immobilized suspension cultures

**Timing: 2 h**

Prepare FN-adherent and PLL-immobilized suspension cultures and label the cells with SPY-DNA before live-cell imaging.

8. Trypsinize the synchronized cells using prewarmed Trypsin-EDTA, resuspend them in the complete medium to obtain a single-cell suspension, and determine the cell number.

***Note:*** Trypsinization and transient loss of adhesion may influence cell-cycle progression and mitotic timing. This represents a potential limitation of the synchronization and imaging procedure.

9. Seed 5 × 10^5^ cells into each FN- or PLL-coated well and incubate for 1 h at 37°C and 5% CO_2_ to allow cells to attach to the FN-coated wells and become immobilized on PLL-coated wells.

***Note:*** The compatibility of plastic culture dishes depends on the microscope objective used. If the selected objective is not compatible with plastic culture vessels, use glass-bottom dishes for live-cell imaging.

10. Add SPY-DNA directly to 3 ml of the complete medium in each well to achieve a final 1× working concentration (1:1000 dilution of the stock solution).
11. Incubate the cells for 1 h at 37°C and 5% CO_2_. This 1 h incubation allows SPY-DNA labelling before image acquisition.

***Note:*** Do not wash out SPY-DNA before imaging; the dye remains in the imaging medium throughout the time-lapse acquisition. The timing was selected to enrich for cells progressing towards mitosis while allowing sufficient time for cell attachment on coating plates before imaging. Protect the culture plates from light after the dye addition.

**CRITICAL:** Seed the same number of cells into FN-adherent and PLL-immobilized suspension cultures to facilitate direct comparison of mitotic progression. For automated time-lapse imaging, place the FN- and PLL-coated wells adjacent to one another to minimize motorized-stage travel during image acquisition.

### Time-lapse live-cell imaging

**Timing: 12–24 h**

Acquire phase-contrast and SPY-DNA fluorescence images under stable environmental conditions to capture mitotic progression over time.

12. Acquire phase-contrast and SPY-DNA fluorescence images according to the imaging procedure.

***Note:*** The imaging system used in this protocol consisted of an inverted Nikon microscope (Eclipse Ti-U) equipped with an Andor Zyla 5.5 PLUS sCMOS camera and a Lumencor SOLA SE fluorescence light source. SPY-DNA fluorescence was acquired using a GFP filter set (ex 472/30, dm 495, em 520/35). Images were acquired at 8-bit depth using an S Plan Fluor ELWD 20X Ph1 ADM objective (NA 0.45), with 1 × 1 camera binning and as single optical planes at 15-min intervals. The image calibration was 0.33 μm/pixel and the camera gain was set to 1 and maintained consistently throughout image acquisition. Exposure settings were optimized before acquisition and maintained consistently throughout each time-lapse imaging.

13. Using NIS-Elements AR or equivalent microscopy software and the motorized stage, define imaging positions in areas containing a sufficient number of cells in each FN- and PLL-coated well.

***Note:*** NIS-Elements AR or equivalent microscopy software can be used for image acquisition and analysis. Z-stack acquisition was not used during time-lapse acquisition.

14. For each position, establish the optimal focal Z-position during the initial setup and maintain the defined focal Z-position throughout the time-lapse acquisition.
15. Acquire phase-contrast and SPY-DNA fluorescence images at each selected imaging position every 15 min for 12–24 h.

***Note:*** Maintain the imaging chamber (Okolab environmental chamber) at 37°C and 5% CO_2_ throughout image acquisition.

16. Set the fluorescence exposure time to maximize the SPY-DNA signal while minimizing background fluorescence and photobleaching.

***Note:*** Determine the exposure settings using adherent cells and apply the same imaging parameters to both adherent and suspension cultures throughout the experiment.

**CRITICAL:** Verify that all selected imaging positions are in focus before initiating time-lapse acquisition. Use identical exposure time, illumination intensity, camera gain, and other acquisition settings for adherent and PLL-immobilized suspension cells to enable direct quantitative comparison of fluorescence intensity.

### ROI tracking and SPY-DNA fluorescence intensity analysis

**Timing: Variable**

Track individual cells and quantify SPY-DNA fluorescence intensity over time to identify mitotic entry, exit, and duration.

17. Export images, separate the channels, and generate surface plots.
  a. Export the phase-contrast and SPY-DNA fluorescence image sequences from NIS-Elements AR.
  b. Separate the corresponding channels and generate a three-dimensional fluorescence-intensity surface plot from the SPY-DNA fluorescence channel (Figures 2, 3, and 4, Methods video S1-Adherent and Methods video S2-Suspension).

**Figure 2:**
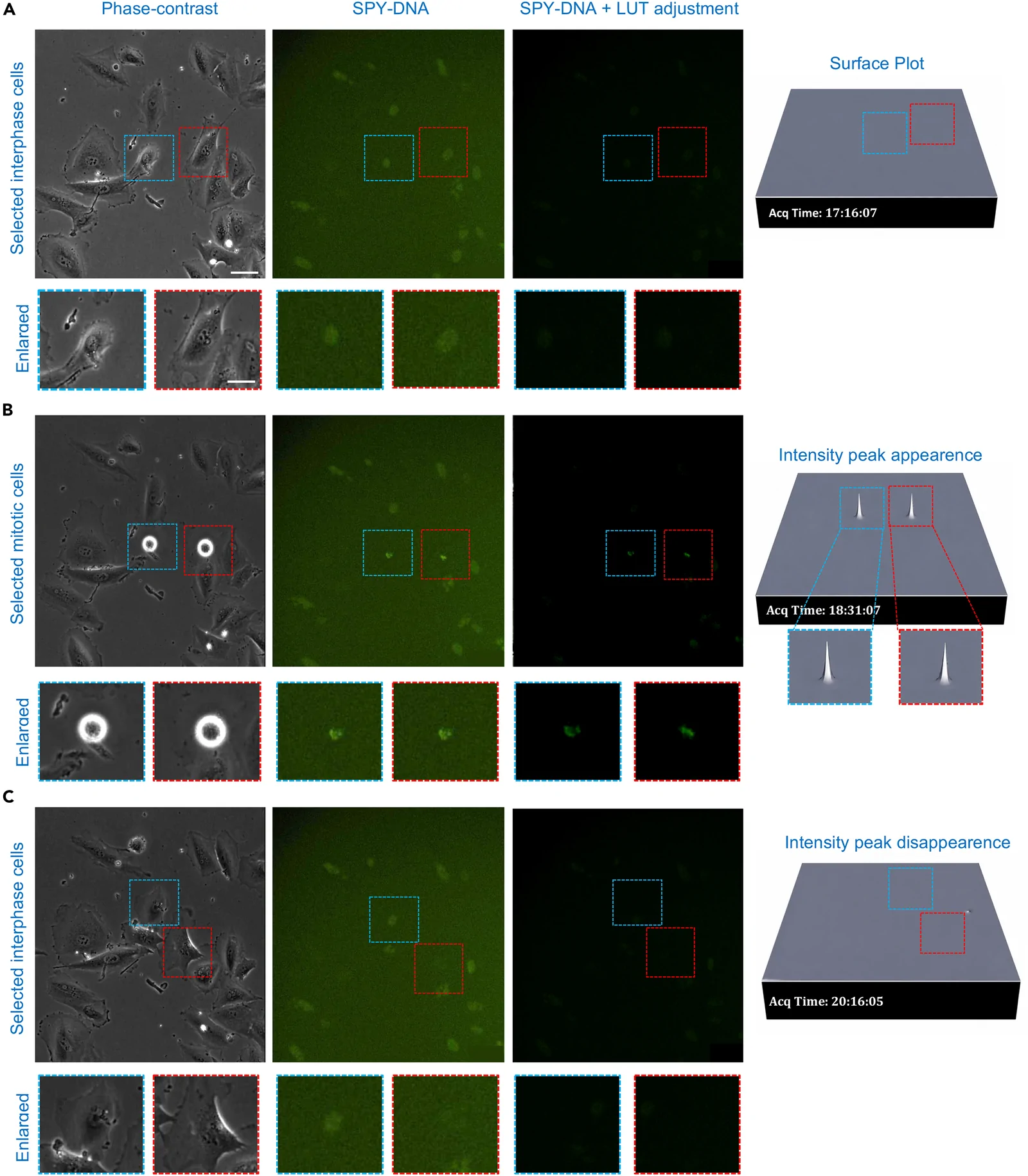
Identification of mitotic entry and exit by SPY-DNA fluorescence and fluorescence intensity surface plot analysis in FN-adherent BJ fibroblasts (A) Representative phase-contrast, SPY-DNA fluorescence, SPY-DNA fluorescence with LUT adjustment, and corresponding fluorescence intensity surface plots of FN-adherent BJ fibroblasts during interphase, mitotic entry, and mitotic exit. During interphase, diffuse chromatin produces a relatively low and dispersed SPY-DNA fluorescence signal without a prominent intensity peak in the surface plot. (B) Representative images of selected cells during mitotic entry. At mitotic entry, chromatin condensation generates a bright, compact SPY-DNA signal accompanied by a distinct fluorescence intensity peak in the surface plot. (C) Representative images of selected cells following mitotic exit. Chromatin decondensation is accompanied by a decrease and disappearance of the fluorescence intensity peak. Enlarged images below each panel highlight the selected cells analyzed at each stage. The acquisition times (Acq Time) displayed in the surface plots indicate the time points of the corresponding representative frames extracted from the time-lapse movie of FN-adherent cells. Scale bars, 50 μm (main images) and 25 μm (enlarged images).

**Figure 3:**
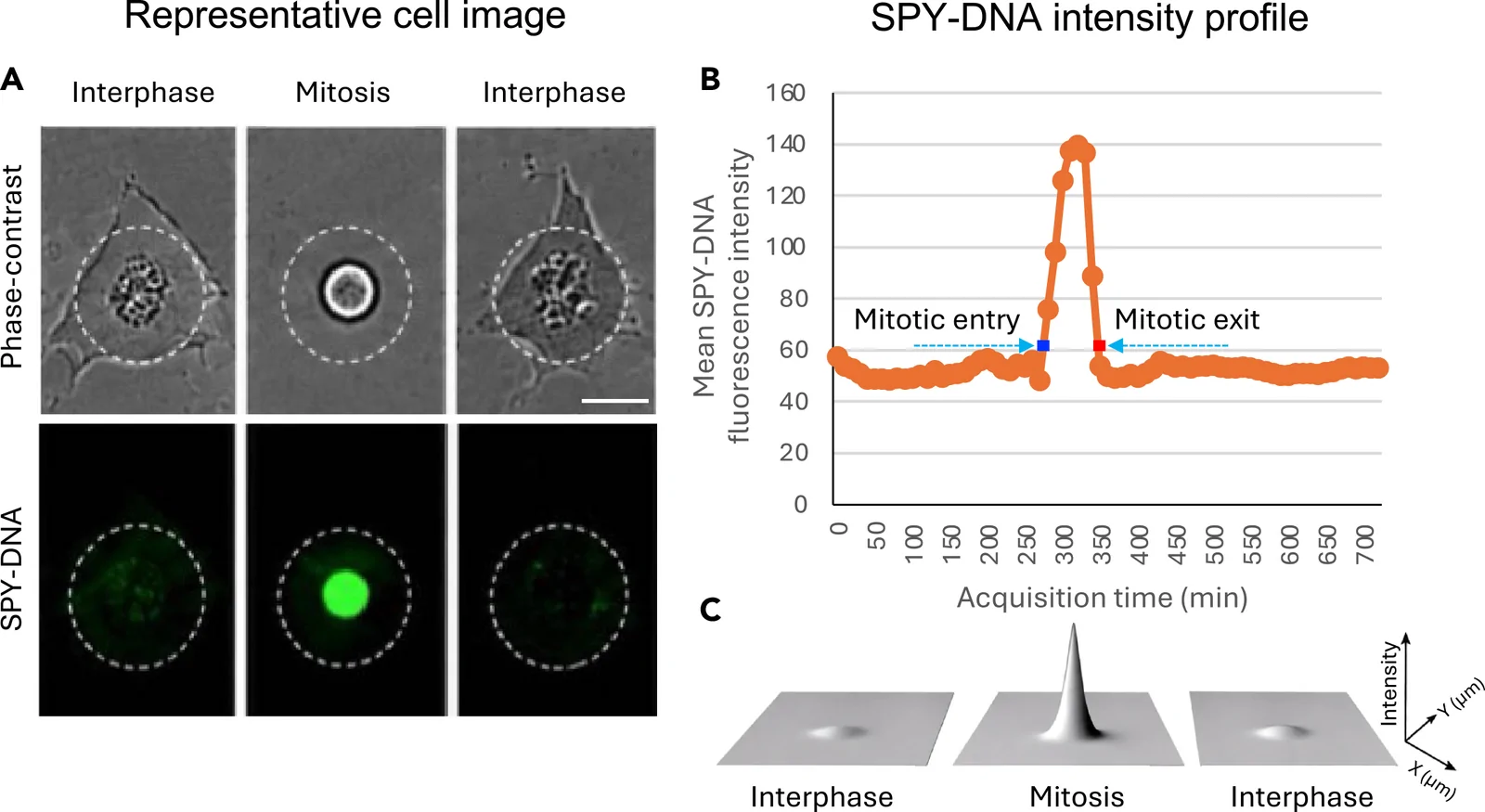
Identification and measurement of mitotic duration using SPY-DNA fluorescence intensity profiles in FN-adherent BJ fibroblasts (A) Representative phase-contrast and SPY-DNA fluorescence images of a BJ fibroblast during interphase, mitotic progression, and the subsequent return to interphase following mitotic exit. The dashed circles indicate the regions of interest (ROI) used for fluorescence intensity analysis. Scale bar, 20 μm. (B) Representative mean SPY-DNA fluorescence intensity profile of a single cell over time. Mitotic entry was identified by an increase in SPY-DNA fluorescence intensity associated with chromatin condensation, whereas mitotic exit was identified by the subsequent decrease in fluorescence intensity following chromatin decondensation. Arrows indicate the start and end points of the mitotic interval on either side of the fluorescence peak. Mitotic entry was defined as the point at which fluorescence intensity increased above the background/interphase level, whereas mitotic exit was defined as the point at which fluorescence intensity returned to the background/interphase level. The time interval between mitotic entry and mitotic exit was used to determine mitotic duration. (C) Schematic representation of the changes in SPY-DNA fluorescence intensity surface profiles during the transition from interphase to mitosis and back to interphase. Interphase is characterized by a relatively low and diffuse fluorescence intensity profile, whereas mitotic chromatin condensation produces a sharp, prominent intensity peak.

**Figure 4:**
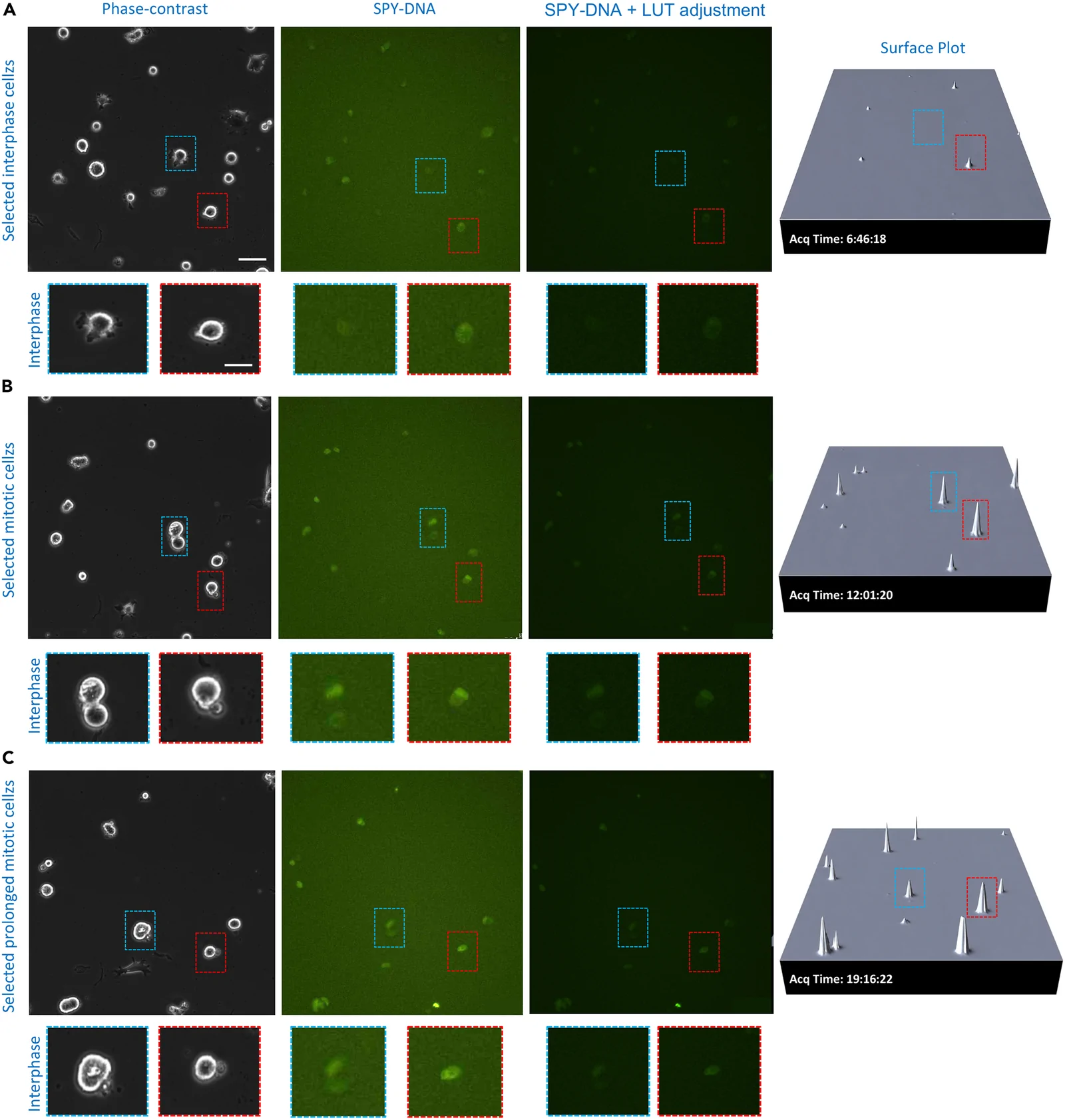
Identification of mitotic progression and prolonged mitosis in PLL-immobilized (suspension) BJ fibroblasts Representative phase-contrast and SPY-DNA fluorescence images, SPY-DNA images with LUT adjustment, and corresponding fluorescence intensity surface plots of hTERT-immortalized BJ fibroblasts cultured on PLL-coated plates under anchorage-deprived conditions during interphase, mitosis, and the subsequent return to interphase. (A) Selected interphase cells, (B) selected mitotic cells, and (C) selected cells undergoing prolonged mitosis are shown. Two representative cells (blue and red boxes) are identified in each panel and shown as enlarged images. During mitosis, chromatin condensation is associated with increased and more concentrated SPY-DNA fluorescence, producing distinct fluorescence intensity peaks in the corresponding surface plots. Cells undergoing prolonged mitosis show sustained elevated SPY-DNA fluorescence intensity and persistence of the fluorescence intensity peak over an extended period. Enlarged images highlight the selected cells analyzed at each stage. Representative acquisition times from the time-lapse recording are indicated on the corresponding surface plots. Scale bars, 50 μm (main images) and 25 μm (enlarged images).
18. Select cells, draw and reposition multiple circular regions of interest (ROIs).
  a. Select individual cells on phase-contrast images at the initial time point by manually creating a ROI around each selected cell, covering the nuclear area, using the Annotation and Measurement tools in ROI Statistic section on NIS-Elements (Figure 3; Figure S1).
  b. Reposition the ROI manually during the time-lapse when necessary to follow lateral cell movement.

***Note:*** The ROI is not resized specifically to follow changes in cell size or chromatin morphology.

**CRITICAL:** Keep the ROI large enough to encompass the cell nuclear area and its chromatin signal.

19. Define background ROI. Place one ROI in a cell-free area and designate it as the background ROI by right-clicking on the ROI.

***Note:*** NIS-Elements automatically subtracts the background fluorescence signal from the cellular ROI measurements, and the resulting values are used for analysis (Figure S1).

20. Adjust LUT.
  a. Adjust the LUT display range to clearly visualize the fluorescence intensity peak corresponding to condensed chromatin and eliminate the background signal in the surface plot.
  b. Keep the same LUT display settings throughout the image analysis for both adherent and suspension conditions (Figures 2, 3, and 4).
21. Identify mitotic entry and exit.
  a. Play the time-lapse sequence and determine the time points of fluorescence peak appearance and disappearance by examining the fluorescence-intensity profiles together with the corresponding phase-contrast images, fluorescence images, and surface plots.
  b. Exclude cells that move substantially out of the focal plane or imaging field, whose fluorescence signal can no longer be reliably assigned to the selected cell, or that overlap with other cells.

***Note:*** Cells displaying irregular or ambiguous fluorescence profiles that do not allow reliable identification of mitotic entry and exit are also excluded from the analysis.

22. Obtain the mean SPY-DNA fluorescence intensity for each ROI using the Measurement/Intensity analysis function in ROI statistics section in NIS-Elements for all images from the initial time point to the end of the movie (Figure 3).

***Note:*** All cell tracking in this protocol was performed manually. Automated cell-tracking tools could be incorporated into future applications of the workflow to facilitate cell tracking and reduce analysis time.

23. Export intensity values.
  a. Export the ROI-associated fluorescence-intensity measurements (mean intensity for the green SPY-DNA channel) as a CSV file.
  b. Plot the mean fluorescence intensity against acquisition time for each ROI to generate individual mitotic profiles (Figure S1).
24. Calculate mitotic duration.
  a. Confirm that the fluorescence-intensity peaks in the line plot correspond to the peaks observed in the surface plots.
  b. Determine the time points at which each peak begins and returns to the background level.
    ***Note:*** Because images are acquired at 15-min intervals, the exact timing of events occurring between consecutive frames cannot be resolved, and mitotic entry, exit, and duration have a temporal uncertainty of up to one imaging interval.
  c. Identify mitotic entry as the first frame in which the mean fluorescence intensity rises above the background level and forms a distinct peak associated with chromatin condensation (Figure 3).
  d. Identify mitotic exit as the first frame in which the mean fluorescence intensity declines back to the background level following chromatin decondensation (Figure 3).
    ***Note:*** The fluorescence-based measurement identifies chromatin condensation and subsequent chromatin decondensation rather than nuclear-envelope breakdown, cell rounding, or chromosome segregation as independent landmarks. Mitotic entry is therefore defined by the onset of detectable chromatin condensation, and mitotic exit by the return of the fluorescence signal to the background level following chromatin decondensation.
  e. Count each distinct fluorescence peak as one mitotic event and calculate mitotic duration using the following equation: Mitotic duration (min) = Mitotic exit time (min) − Mitotic entry time (min).
    ***Note:*** In the adherent condition, the appearance and disappearance of the fluorescence peak are consistent with the characteristic cell rounding and subsequent return toward an interphase morphology associated with mitotic entry and exit, respectively (Figure 3).
    **CRITICAL:** Establish the LUT display range, surface plot display setting, ROI size, and fluorescence intensity measurement settings using adherent cells, where mitotic progression can be evaluated by both cell morphology and chromatin condensation. Apply identical analysis parameters to suspension cells to minimize analysis bias and to enable direct quantitative comparison between culture conditions.

Methods video S1. Adherent: Live-cell imaging of mitotic progression in FN-adherent cells (related to step 16) Representative time-lapse sequence of phase-contrast images, SPY-DNA fluorescence images, and corresponding SPY-DNA fluorescence intensity surface plots of BJ fibroblasts cultured on FN-coated plates during mitotic progression.

Methods video S2. Suspension: Live-cell imaging of mitotic progression in PLL-immobilized (suspension) cells (related to step 16) Representative time-lapse sequence of phase-contrast images, SPY-DNA fluorescence images, and corresponding SPY-DNA fluorescence intensity surface plots of BJ fibroblasts cultured on PLL-coated plates under suspension conditions during mitotic progression.

## Expected outcomes

Successful execution of this protocol generates high-quality phase-contrast and SPY-DNA fluorescence time-lapse image sequences that enable quantitative analysis of mitotic progression in both adherent and suspension cells. During interphase, SPY-DNA produces a diffuse nuclear fluorescence signal, whereas chromatin condensation at mitotic entry generates a bright, compact fluorescence signal. At mitotic exit, the fluorescence intensity decreases as the condensed chromatin decondenses, marking the end of the measured mitotic interval. Surface plot visualization of SPY-DNA fluorescence generates a distinct fluorescence intensity peak during mitosis, whereas interphase cells display a relatively flat intensity profile. Measurement of the mean SPY-DNA fluorescence intensity over time produces a characteristic intensity profile that enables quantitative identification of mitotic entry, mitotic exit, and mitotic duration for individual cells (Figure 3). Using this workflow, FN-adherent and PLL-immobilized suspension cells can be analyzed under identical imaging and analysis conditions, enabling direct comparison of mitotic progression between the two culture conditions. In the representative experiments described here, PLL-immobilized suspension cells showed longer mitotic durations than FN-adherent cells, reflected by prolonged persistence of the fluorescence intensity peak before chromatin decondensation (Figure S2).

Although this protocol was developed and validated using non-transformed BJ fibroblasts, it could potentially be adapted to anchorage-independent cancer cells. Many cancer cells can proliferate efficiently under suspension conditions,^8^ therefore, this workflow could potentially be adapted for quantitative comparison of mitotic progression between normal and transformed cells, subject to validation in the relevant cell lines. The protocol generates time-resolved fluorescence profiles for individual cells and allows calculation of mitotic entry, mitotic exit, and mitotic duration. For each experimental condition, report the total number of cells analyzed, the number of independent biological experiments, the number and percentage of cells entering and completing mitosis, and the number and reasons for excluded cells. Mitotic duration should be presented as individual-cell measurements, together with summary statistics for each independent biological experiment.

## Limitations

This protocol quantifies mitotic progression by monitoring chromatin condensation and decondensation using SPY-DNA fluorescence. Although this approach provides a fluorescence intensity-based, morphology-independent method for identifying mitotic entry and mitotic exit, its performance depends on the fluorescence properties of the DNA dye, including signal intensity, photostability, and specificity for chromatin. Future improvements in live-cell DNA probes with increased brightness, higher photostability, and lower phototoxicity may further enhance the sensitivity and enable longer-duration live-cell imaging experiments.

This protocol focuses on chromatin dynamics and does not directly visualize other cellular structures involved in mitosis. Combining SPY-DNA with spectrally distinct live-cell probes, such as SPY-Tubulin^9^ (Figure S2) or SPY555-Actin,^2^^,^^10^ may provide complementary information on spindle assembly, chromosome segregation, and cytoskeletal dynamics during mitotic progression. When used in combination, the second probe can be added at the same time as the SPY-DNA dye and detected using spectrally distinct excitation and emission wavelengths to allow simultaneous imaging of both signals.

Successful implementation of the protocol depends on efficient cell synchronization and stable live-cell imaging. Incomplete synchronization decreases the proportion of cells entering mitosis during image acquisition, reducing experimental efficiency. In addition, prolonged fluorescence imaging may cause photobleaching or phototoxicity if imaging intervals are too short or excitation intensity is too high.

Although PLL effectively immobilizes cells under the anchorage-deprived condition during imaging, occasional cell displacement may still occur, particularly during long-term acquisitions or frequent handling of the culture plate.

Finally, the timing of mitotic progression varies among different cell types and experimental conditions. Therefore, synchronization conditions, SPY-DNA labeling, and imaging parameters may require optimization before applying this protocol to additional cell models.

## Troubleshooting

### Problem 1

Weak or inconsistent SPY-DNA fluorescence signal (Step 10–12).

### Potential solution

• Prepare the SPY-DNA working solution at the concentration specified in the protocol and use it freshly prepared.
• Protect the dye and labeled cells from light throughout the experiment.
• Confirm that the microscope excitation and emission settings are appropriate for SPY-DNA.
• If photobleaching is observed, reduce the excitation intensity or increase the imaging interval.
• If the fluorescence signal remains weak, optimize the SPY-DNA concentration and incubation time for the cell type being studied while minimizing background fluorescence and phototoxicity.
• If required, alternative live-cell DNA probes with different spectral properties, such as SPY555-DNA^11^ (Excitation/Emission: 555/580 nm), may be used after optimizing the corresponding imaging parameters.

### Problem 2

Low percentage of mitotic cells during live-cell imaging (Steps 2–5).

### Potential solution

• Confirm that the double thymidine block is performed using the recommended thymidine concentration and incubation times.
• Verify that the cells are actively proliferating before synchronization and are maintained under optimal culture conditions.
• Avoid over-confluent or under-confluent cultures, as both can reduce synchronization efficiency.
• Use freshly prepared thymidine solutions to ensure optimal synchronization efficiency.
• If necessary, optimize the synchronization conditions for the cell line of interest, as different cell types may respond differently to double thymidine block.
• For slowly proliferating cell lines or cells that are difficult to synchronize, serum starvation followed by double thymidine block may be considered as an alternative approach, but this should be validated for the specific cell type because additional starvation may introduce cellular stress and alter cell-cycle behavior.

### Problem 3

Suspension cells move during time-lapse imaging.

### Potential solution

• Confirm that the PLL coating has been prepared according to the manufacturer’s instructions.
• Minimize motorized stage movement by selecting imaging positions in adjacent wells and reducing stage travel whenever possible.
• If supported by the microscope software, reduce the motorized stage movement speed to minimize vibrations during image acquisition.
• Allow the cells to become immobilized on the PLL-coated surface for approximately 1 h before starting time-lapse imaging.
• Avoid prolonged incubation of cells on PLL-coated plates during image acquisition, as extended incubation may increase cell attachment and alter the suspension phenotype.
• Coating ultra-low-attachment plates with PLL may improve immobilization of suspension cells during long-term time-lapse imaging.

### Problem 4

Loss of focus during long-term imaging (Steps 12–14).

### Potential solution

• Allow the environmental chamber to reach stable temperature and CO_2_ conditions before imaging.
• Establish and verify the optimal focal Z-position at each imaging position before starting the time-lapse acquisition.
• Reduce unnecessary movement of the microscope stage during image acquisition.

### Problem 5

Fluorescence intensity peaks are poorly defined in surface plots (Steps 14–15).

### Potential solution

• Use a circular ROI that encompasses the selected cell nuclear area while excluding neighboring cells.
• Adjust the LUT display range before generating the surface plot to improve visualization of the fluorescence intensity peak associated with condensed chromatin while minimizing visibility of the background signal.
• Verify that cells remain in focus throughout the time-lapse sequence, as slight focus drift can reduce peak intensity.
• Exclude cells with incomplete image sequences, overlapping nuclei, excessive background fluorescence, or substantial movement out of the imaging field.
• Confirm that fluorescence exposure time is sufficient to generate a well-defined fluorescence peak without saturating the detector.

### Problem 6

Photobleaching or phototoxicity during long-term imaging.

### Potential solution

• Reduce the excitation light intensity whenever possible while maintaining sufficient fluorescence signal.
• Increase the imaging interval if compatible with the experimental design.
• Minimize the exposure time while maintaining an adequate signal-to-noise ratio.
• Protect SPY-DNA-labeled cells from ambient light before and during image acquisition.
• Maintain the camera gain at 1 and minimize illumination intensity while maintaining sufficient fluorescence signal.
• Optimize the SPY-DNA concentration for the cell type being studied to maximize fluorescence while minimizing phototoxicity.
• Keep environmental conditions (37°C and 5% CO_2_) stable throughout long-term imaging to reduce cellular stress.

## Resource availability

### Lead contact

Further information and requests for resources and reagents should be directed to and will be fulfilled by the lead contact, Siamak A. Kamranvar (kamranvars@cardiff.ac.uk).

### Technical contact

Technical questions regarding the execution of this protocol should be directed to and will be answered by the technical contact, Siamak A. Kamranvar (kamranvars@cardiff.ac.uk).

### Materials availability

This protocol did not generate any unique reagents, cell lines, plasmids, or other materials. All reagents and resources are commercially available and listed in the key resources table.

### Data and code availability

This protocol does not include custom code. The raw data supporting Figure S1 are provided as a supplementary Excel file. Data underlying Figure S2 were previously published in Kamranvar et al.^1^ Additional information related to image analysis is available from the lead contact upon reasonable request.

## Acknowledgments

This work was primarily carried out at Uppsala University, with additional work conducted at Cardiff University. Live-cell imaging was performed using the microscopy facility at the IMBIM Department, Uppsala University. The author thanks Prof. Staffan Johansson for his valuable support, insightful discussions, and continuous encouragement during the development of this protocol. This work was supported by the 10.13039/100012538Swedish Cancer Foundation (Cancerfonden; grant no. 190531Pj, awarded to Prof. Staffan Johansson) and by the 10.13039/100010269Wellcome Trust Career Development Award (grant no. 226510/Z/22/Z), awarded to Dr. Greg Ngo.

## Author contributions

S.A.K. conceived and designed the protocol, performed the experiments, analyzed the data, and wrote the manuscript.

## Declaration of interests

The author declares no competing interests.
