# Supplementary material for: Protocol for quantifying mitotic progression in adherent and suspension cells by live-cell imaging
Source: STAR Protoc. 2026 Oct 13;7(4):104899. doi: 10.1016/j.xpro.2026.104899 (PMC13686043; doi:10.1016/j.xpro.2026.104899)
Supplement: Supplementary file 1 [file mmc1.pdf]

Supplementary figure 1

A

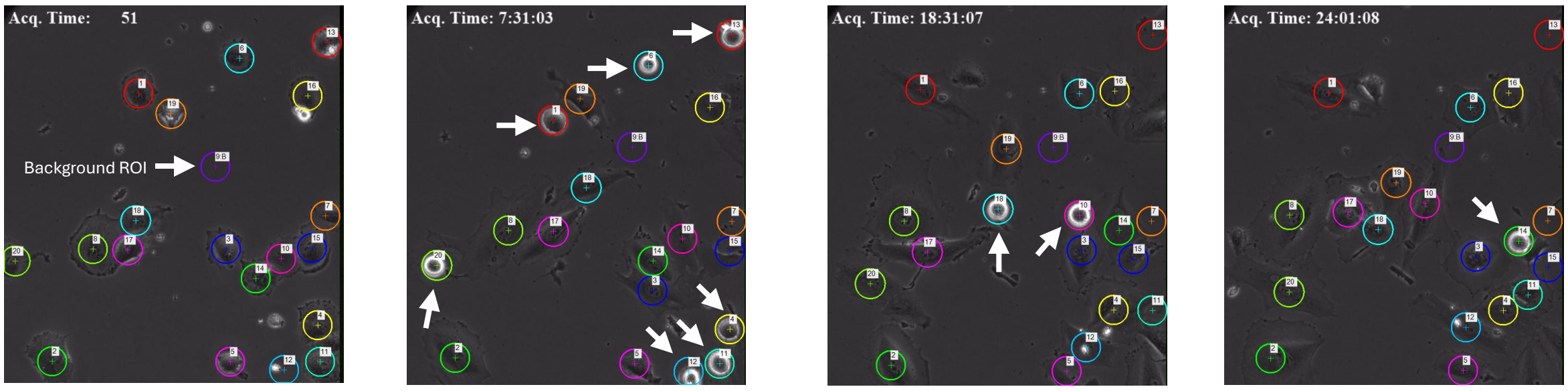

B

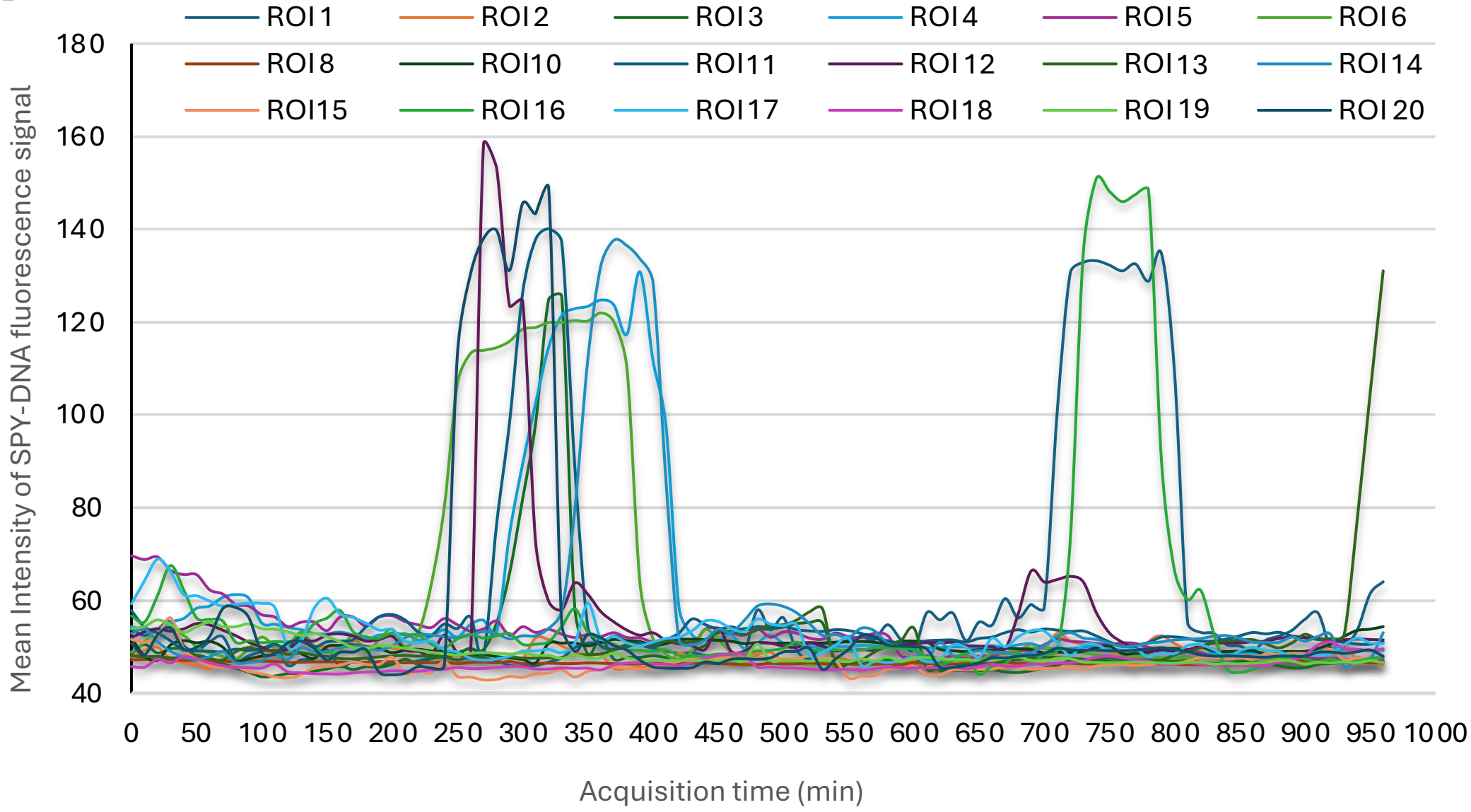

Supplementary Figure 1. Workflow for analysis of SPY-DNA fluorescence intensity in time-lapse movies, related to steps 18-23.
(A) Representative images from a time-lapse movie showing individual cells selected for analysis. Regions of interest (ROIs) were manually drawn around the selected cell/nuclear area of each selected cell using the ROI Statistics function in NIS-Elements. Each colored ROI represents an individual cell followed throughout the time-lapse movie. An additional ROI was placed in a cell-free area to measure background fluorescence. This background signal was automatically subtracted by NIS-Elements from the fluorescence intensity measurements of the cellular ROIs. Arrows indicate examples of cells undergoing mitosis during the time-lapse sequence, except for an arrow in the first image on the left, which indicates the background ROI. (B) Background-subtracted mean SPY-DNA fluorescence intensity of individual cells plotted throughout the time-lapse movie. Mitotic entry was identified by an increase in SPY-DNA fluorescence intensity above the background level, corresponding to chromatin condensation, whereas mitotic exit was identified by the subsequent decrease in fluorescence intensity to the background level following chromatin decondensation. Each distinct fluorescence intensity peak was considered a mitotic event, allowing the timing and number of mitotic events to be determined for individual cells.

Supplementary figure 2

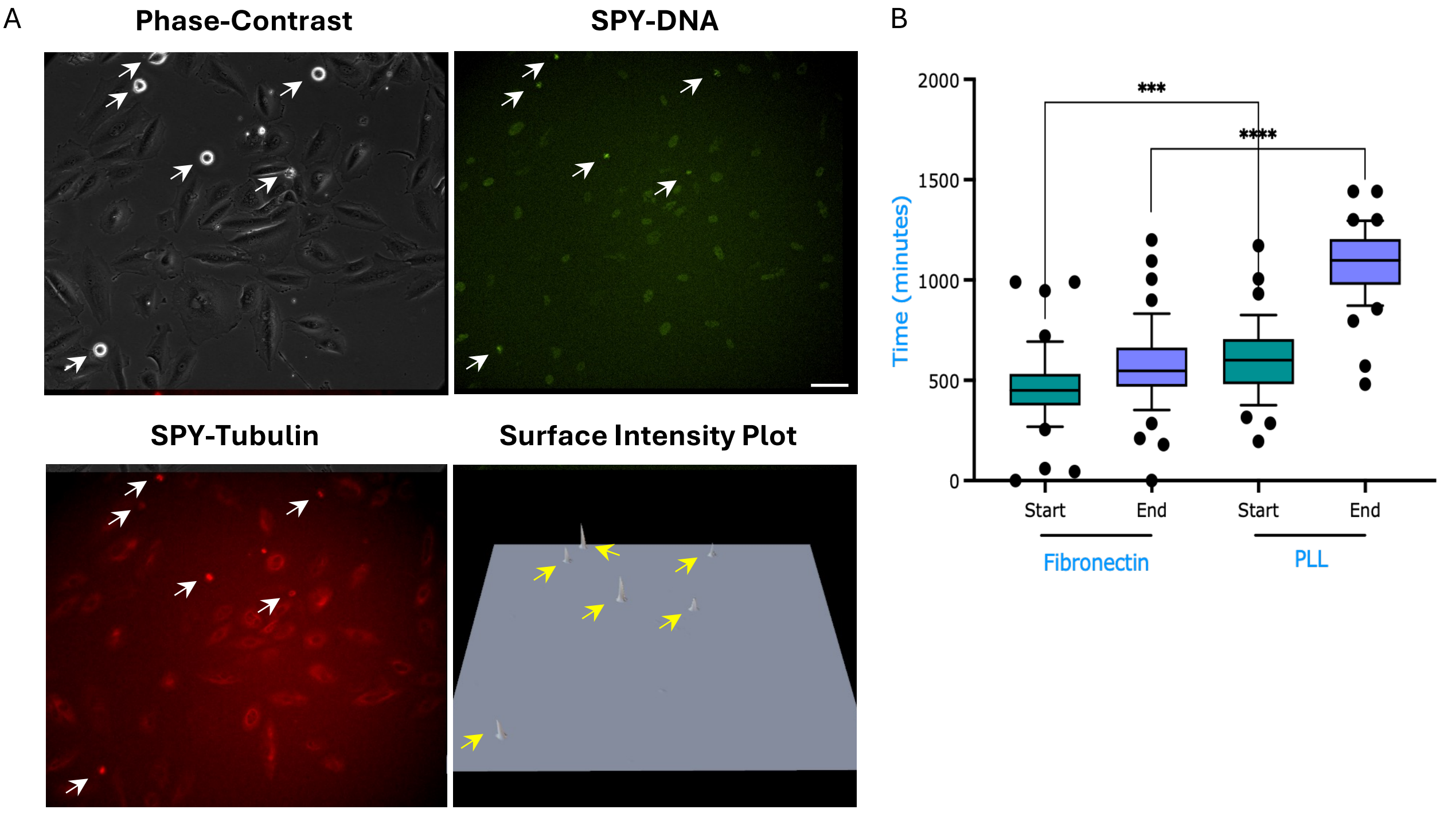

Supplementary Figure 2. Additional support for SPY-DNA-based identification of mitotic cells and comparison of mitotic entry and exit times under FN-adherent and PLL-immobilized suspension conditions, related to steps 24.

(A) Representative phase-contrast, SPY-DNA, and SPY-Tubulin fluorescence images of cells under the indicated culture conditions. Arrows indicate representative cells undergoing mitosis. Chromatin condensation detected by SPY-DNA is accompanied by a bright, compact fluorescence signal and corresponding fluorescence intensity peaks in the SPY-DNA fluorescence intensity surface plot. Mitotic cells identified by SPY-DNA fluorescence also show corresponding changes in SPY-Tubulin fluorescence, providing additional support that the observed SPY-DNA fluorescence intensity peaks are associated with mitotic progression. (B) Comparison of mitotic entry and exit times in cells cultured on FN-adherent and PLL-immobilized conditions. Mitotic duration can be determined as the time interval between mitotic entry and mitotic exit based on changes in mean SPY-DNA fluorescence intensity. The box plots show the distribution of mitotic entry and exit times. Statistical significance is indicated as ***$p < 0.001$ and ****$p < 0.0001$. The data shown in this panel were previously published in Kamranvar et al.[1] The figure was recreated using the same underlying dataset.
